# Ghrelin in Chronic Kidney Disease

**DOI:** 10.1155/2010/567343

**Published:** 2010-03-17

**Authors:** Wai W. Cheung, Robert H. Mak

**Affiliations:** Division of Pediatric Nephrology, Department of Pediatrics, University of California San Diego, 9500 Gilman Drive, Mail code no. 0634, La Jolla, CA 92093-0634, USA

## Abstract

Patients with chronic kidney disease (CKD) often exhibit symptoms of anorexia and cachexia, which are associated with decreased quality of life and increased mortality. Chronic inflammation may be an important mechanism for the development of anorexia, cachexia, renal osteodystrophy, and increased cardiovascular risk in CKD. Ghrelin is a gastric hormone. The biological effects of ghrelin are mediated through the growth hormone secretagogue receptor (GHSR). The salutary effects of ghrelin on food intake and meal appreciation suggest that ghrelin could be an effective treatment for anorexic CKD patients. In addition to its appetite-stimulating effects, ghrelin has been shown to possess anti-inflammatory properties. The known metabolic effects of ghrelin and the potential implications in CKD will be discussed in this review. The strength, shortcomings, and unanswered questions related to ghrelin treatment in CKD will be addressed.

## 1. Introduction

The cachexia syndrome in patients with chronic kidney disease (CKD) consists of muscle wasting, anorexia, and increased elevated energy expenditure. Cachexia is an important risk factor for mortality in patients with CKD, which is 100-fold to 200-fold higher than in the general population. Cachexia, a common feature in many chronic inflammatory diseases, is distinct from malnutrition, which is defined as the consequence of insufficient nutrients [1]. Responses in malnutrition are adaptive, whereas those in cachexia are maladaptive. In malnutrition, such as in simple starvation, fats are preferentially utilized and lean body mass is preserved. In cachexia, muscle mass is wasted and fats are relatively underutilized. Anorexia, defined as the loss of desire for food, is prevalent in patients CKD. Anorexia in CKD patients can arise from decreased taste and smell of food, early satiety, dysfunctional hypothalamic membrane adenylate cyclase, increased brain tryptophan, and increased cytokine production. Anorexia reduces oral energy and protein intakes and contributes to the development of cachexia. Elevated resting energy expenditure was associated with increased mortality and cardiovascular death in CKD and was closely correlated with the prevalence of cachexia among these patients [2]. To date, there is no effective therapy for cachexia in CKD. Nutritional strategies such as caloric supplementation and appetite stimulants have been largely unsuccessful. Thus, there is an urgent need for the development of new therapeutic agents for this potentially fatal complication of CKD [3]. 

## 2. Energy Metabolism in CKD

New insights into the pathophysiolgy of cachexia in CKD hold the promise of novel therapeutic strategies. One important mechanism for cachexia in CKD is that elevated circulating proinflammatory cytokines act on CNS and regulate the release and function of a number of key neuropeptides, thereby influence energy metabolism. Leptin and central melanocortin systems have been suggested as targets of cytokine action in the hypothalamus, which is a critical regulator of food intake and energy homeostasis [4]. There are two distinct subsets of neurons that control food intake in the hypothalamus. One subset of neurons produce neuropeptide Y (NPY) that stimulates food intake while an adjacent neuronal subset produces melanocortin peptides, which inhibit eating. Circulating leptin and insulin decrease appetite by inhibiting NPY and agouti-related peptide (AgRP), while stimulating the production of melanocortin peptides in the hypothalamus. Inflammatory cytokines induce anorexia by their central actions. Cytokines regulate gastrointestinal activities, cause metabolic changes, affect the endocrine system, and modulate the neuropeptide profile of the hypothalamus, all of which can influence eating behavior [1, 5]. We have demonstrated that cachexia in a mouse model of CKD could be ameliorated by genetic or pharmacological blockade of leptin and central melanocortin signaling via the melanocortin receptor type-4 (MC4-R) [6]. However, potential clinical utility of this approach is limited by the need to deliver AgRP intracerebroventricularly. We then examined the effects of NBI-12i, a small molecule MC4-R reverse agonist, in a mouse model of uremic cachexia. Intraperitoneal injection of NBI-12i ameliorated uremic cachexia. The protective effects of NBI-12i may be due to the normalization of the upregulation of uncoupling protein expression seen in CKD mice [7]. These data underscore the importance of melanocortin signaling in the pathogenesis of uremic cachexia and demonstrate the potential utility of MC4-R antagonists as a novel therapeutic approach. 

## 3. Ghrelin Physiology

Ghrelin is a gut peptide that stimulates the production of growth hormone (GH) from the pituitary gland [8]. Ghrelin, a natural ligand of the GH secretagogue receptor 1a (GHS-R1a), is secreted into the bloodstream primarily from the stomach and small intestine. Ghrelin is mainly degraded by the kidney [9]. In addition to stomach and small intestine, small amount of ghrelin has also been detected in hypothalamic arcuate nucleus and many other tissues. GHS-R1a is expressed by neurons in the arcuate nucleus and the ventromedial hypothalamus. Ghrelin is a circulating hunger hormone and is considered the counter regulatory hormone for leptin. Ghrelin levels increase before meal and decrease after meals. Ghrelin and synthetic ghrelin analogues increase food intake by an action exerted at the level of the hypothalamus. They activate cells in the arcuate nucleus that include the orexigenic NPY neurons [10]. Ghrelin-responsiveness of these neurons is both leptin- and insulin-sensitive [11]. Ghrelin also activates the mesolimbic cholinergic/dopaminergic pathways, a circuit that communicates the hedonic and reinforcing aspects of natural rewards, such as food and ethanol [12]. Most studies to date have focused on the effects of pharmacological doses of ghrelin and its analogues in human and animal models. These effects include stimulation of GH-releasing activity, stimulation of ACTH release, inhibition of gonadotropin secretion, stimulation of appetite and positive energy balance, changes in gastric motility and acid secretion, protective effects against gastric mucosal injury, and modulation of pancreatic function and glucose metabolism enhanced cardiovascular performance via GH-growth-like factor (IGF-I) axis, as well as modulation of immune system and bone biology [9, 13, 14].

Three distinguished ghrelin gene products, that is, acyl ghrelin, des-acyl ghrelin and obestatin, have been identified. Acyl ghrelin was identified as the endogenous cognate ligand for the GHS-R1a [8]. The second endogenous cognate ligand for GHS-R, des-Gln14-ghrelin, another novel 27-amino acid peptide, is created by alternative splicing of the ghrelin gene and constitutes one fifth of ghrelin immunoreactivity of the rat stomach [15]. Acyl ghrelin increases meal size [16, 17]. Acyl ghrelin and GHRH are both endogenous GH-releasing peptides. GHRH acts on the GHRH receptor, distinct from GHS-R1a, to activate adenylate cyclase and to increase intracellular cAMP, which serves as a second messenger to activate subsequent signaling cascade [9]. In contrast, des-acyl ghrelin, lacking *O*-*n*-octanoylation at serine 3, is also produced in the stomach and remains the major molecular form secreted into the circulation. Des-acyl ghrelin has been shown to actively participate in food intake [18], gut motility [19], body size development [19, 20], adipogenesis [21], insulin secretion and resistance [22], and to increase tension of guinea pig papillary muscle ex vivo [23], and cell proliferation and survival in vitro [24, 25]. Des-acyl ghrelin was secreted in a highly regulated manner in response to food deprivation in mice [26]. Intracerebroventricular (icv) administration of rat des-acyl ghrelin to rats or mice stimulated feeding and induced the expression of *Fos*, a marker of neuronal activation, in orexin-expressing neurons of the lateral hypothalamic area. Peripheral administration of des-acyl ghrelin to rats or mice did not affect feeding. Des-acyl ghrelin increased the intracellular calcium concentrations in isolated orexin neurons. Central des-acyl ghrelin may activate orexin-expressing neurons, perhaps functioning in feeding regulation through interactions with a target protein distinct from the GHS-R1a [27]. The third ghrelin gene product, obestatin, a novel 23-amino acid peptide identified from rat stomach, was derived from the mammalian prepro-ghrelin gene, which also encodes ghrelin, by comparative genomic analyses [28]. It was originally projected that obestatin binds to an orphan G protein-coupled receptor, termed GPR39, to inhibit food intake [28]. Obestatin induces early-response gene expression in stomach, intestine, white adipose tissue, liver, and kidney, suggesting its role as a gastrointestinal and metabolic hormone [29]. Obestatin activates neurons in several brain regions. Icv injection of obestatin inhibits thirst and vasopressin secretion, suppresses food intake, regulates sleep, decreases anxiety, and improves memory. Coupled with the ability of obestatin to activate cortical neurons and to stimulate the proliferation and downstream signaling of human retinal pigment epithelial cells, these findings underscore diverse functions of obestatin [30]. Disturbance of circulating ghrelin and obestatin may have a role in the pathogenesis of cachexia. Obestatin levels were significantly increased in cardiac patients with cachexia than patients without cachexia and healthy controls [31]. Serum and saliva ghrelin and obestatin levels were elevated in 24 hemodialysis patients compared with age-matched healthy controls [32]. Obestatin manifested various biological functions, such as improving memory performance, causing anxiolytic effects [33], inhibiting thirst in rats [34], activating cortical neurons [35], stimulating proliferation of retinal pigment epithelial cells in vitro [36], and profoundly influencing sleep [37, 38]. 

Serine-3 of ghrelin, which is acylated with an eight-carbon fatty acid, octanoate, is inextricably required for the multifaceted endocrine functions of acyl ghrelin. Despite the crucial role for octanoylation in the physiology of ghrelin, the lipid transferase that mediates this novel modification had remained unknown until recently. In 2008, ghrelin *O*-acyltransferase (GOAT), attaching octanoate to serine-3 of ghrelin, was identified and characterized by two independent research groups [39, 40]. Ghrelin seems to be the sole substrate for GOAT. GOAT is located in the endoplasmic reticulum, and the presumed donor for octanoylation is octanoyl-CoA. Expression of GOAT was demonstrated to be limited to the gastrointestinal tract, intestine, testis [39], and the pancreas [40], the major ghrelin-secreting tissues. The discovery of GOAT, an enzyme specific for octanylation of ghrelin, may hold the promise of answering some major questions about the unknown but important physiological roles of ghrelin [41].

## 4. Ghrelin and Body Composition

Muscle mass is important for physical fitness and metabolic regulation. Human observational study suggested that fasting plasma ghrelin concentration is related to skeletal muscle mass in healthy adults. After accounting for other covariates, total body skeletal mass was a significant negative predictor of ghrelin concentrations [42]. Effects of an oral ghrelin mimetic on body composition and clinical outcomes in 65 healthy older adults were analyzed. Over 12 months, the ghrelin mimetic MK-667 enhanced growth hormone secretion, significantly increased fat-free mass, and was generally well tolerated [43]. In a longitudinal study, relationship between body composition and plasma ghrelin levels was investigated in a group of end-stage renal disease (ESRD) adult patients. Changes in plasma ghrelin during 12 months of peritoneal dialysis treatment are associated with changes in body composition. Markedly elevated plasma ghrelin levels are found in advanced renal failure and correlate with fat mass [44]. Ghrelin regulates fat distribution and energy metabolism in lean tissues such as liver and muscles. In liver, ghrelin induced a lipogenic and glucogenic pattern of gene expression and increased triglyceride content while reducing activated (phosphorylated) stimulator of fatty acid oxidation, AMP-activated protein kinase (AMPK), with unchanged mitochondrial oxidative enzyme activities. In contrast, triglyceride content was reduced after ghrelin administration in mixed (gastrocnemius) and unchanged in oxidative (soleus) muscle. In mixed muscle, ghrelin increased mitochondrial oxidative enzyme activities independent of changes in expression of fat metabolism genes and phosphorylated AMPK. Expression of peroxisome proliferator-activated receptor-*γ* (PPAR-*γ*), the activation of which reduces muscle fat content, was selectively increased in mixed muscle where it paralleled changes in oxidative capacities [45]. Thus ghrelin induces tissue-specific changes in mitochondrial and lipid metabolism gene expression and favors triglyceride deposition in liver over skeletal muscle. These novel effects of ghrelin in the regulation of lean tissue fat distribution and metabolism could contribute to metabolic adaptation to caloric restriction and loss of body fat. 

## 5. Ghrelin and Inflammation

Chronic inflammation modulates ghrelin levels in humans and rats [46]. In rat model of adjuvant-induced arthritis, there is a compensatory variation of ghrelin levels that relates to body weight adjustments [46]. Recovery of ghrelin levels in the latter stage suggests an adaptive response and may represent a compensatory mechanism under catabolic conditions. Similar results were observed in patients with rheumatoid arthritis [46]. Recent studies also suggest that prostacyclin signaling regulates circulating ghrelin during acute inflammation. Madison et al. have investigated the mechanism of the regulation of ghrelin by inflammation. Ghrelin levels fall in states of acute inflammation brought about by injection of bacterial lipopolysaccharide (LPS). They demonstrate that IL-1*β* receptor is expressed within the gastric mucosa, but is not expressed by ghrelin cells. The prostacyclin receptor was also expressed in the gastric mucosa, and the majority of ghrelin-producing cells were found to co-express this receptor. Mice with genetic deletion of the IL-1*β* receptor do not suppress circulating ghrelin levels with LPS administration [47]. Collectively, their data support the notion that inflammation-induced decreases in ghrelin are likely due to the action of IL-1*β* on cells within the gastric mucosa that in turn produce prostacyclin as a second messenger. These data provide further support for the potential role of ghrelin as a therapeutic agent in acute and chronic inflammatory diseases.

## 6. Ghrelin and CKD

Conflicting results of circulating ghrelin levels in CKD have been presented. Elevated plasma ghrelin levels were observed in adult dialysis patients than those of age-matched controls [48]. Szczepańska et al. reported that plasma ghrelin levels were similar in CKD children on dialysis compared with children on conservative treatment and healthy controls [49]. In another study, adult hemodialysis patients showed similar serum ghrelin levels whereas peritoneal dialysis patients exhibited significantly lower serum ghrelin concentrations than predialysis CKD patients [50]. Multiple confounding factors may contribute to these seemingly contradicting findings. The two major forms of circulating ghrelin are acylated (<10%) and des-acyl ghrelin [15, 51]. Acylated ghrelin promotes food intake while des-acyl ghrelin induces negative energy balance. Most of the investigators have used the traditional radioimmunoassay method to analyze the sum of both acylated and des-acyl ghrelin. However, only plasma des-acyl ghrelin levels were elevated in CKD patients compared with those patients with normal renal functions. Recent studies have suggested that elevated des-acyl ghrelin levels could be involved in the anorexia on CKD patients [52]. Age and sex influence plasma ghrelin levels. Plasma ghrelin levels were negatively correlated with age in CKD patients [48]. Significantly higher total ghrelin levels were observed in female subjects [53]. Variations in residual renal function affect ghrelin metabolism. The kidney degrades ghrelin. Increased total ghrelin levels in CKD are primarily due to the decreased degradation of ghrelin in the kidney [52]. Nutritional status of CKD patients may also influence the metabolism of ghrelin. Assessment of nutritional status has not been performed in most studies in which chronic energy deficiency is evident in CKD patients. In addition, nutrients may be introduced during dialysis in CKD patients. The absorption of glucose and other nutrients such as amino acids may influence plasma ghrelin levels [50, 54]. Longitudinal studies, by following the same patients cohort in terms of their serum ghrelin and nutritional status [44], and by applying the discriminating ELISA assays to differentiate acylated versus des-acyl ghrelin levels, are more likely to reveal the pathophysiologic role of ghrelin in CKD. 

Skeletal muscle mitochondrial dysfunction [55] and insulin resistance [56, 57] have been reported in CKD and may contribute to metabolic and cardiovascular morbidity and mortality in advanced CKD patients. Administration of ghrelin enhanced muscle mitochondrial enzyme activities and AKT-mediated insulin signaling independently of food intake in healthy rats [45, 58]. Consistent with these findings, a direct association was also shown between insulin-mediated glucose metabolism and plasma ghrelin concentrations in nondiabetic uremic patients undergoing maintenance hemodialysis [59]. Effects of ghrelin in skeletal muscle mitochondrial oxidative capacity and AKT phosphorylation in a rat model of CKD were investigated. Results indicated that ghrelin administration attenuated CKD-associated reduction of mitochondrial oxidative capacity, as reflected by representative mitochondrial enzyme activated. These changes were associated with a higher transcriptional expression of mitochondrial biogenesis stimulators such as peroxisome proliferators activated receptor-*γ*-coactivator-1*α* (PGC-1*α*) and -*β* (PGC-1*β*) [60]. Thus, effects of ghrelin on appetite and muscle mitochondria may improve muscle metabolic and nutritional alternations in CKD. 

## 7. Ghrelin and Its Analogues as Potential Therapeutic Agents for CKD-Associated Cachexia

There is an urgent need for effective appetite-stimulatory therapies for CKD patients. Ghrelin is more potent than any other orexigenic factors as it rapidly enhances food consumption following injection in rodents [61] and humans [62]. Results of recent findings bolster the potential therapeutic application of ghrelin and its analogues as an appetite stimulating and anabolic strategy in uremia-associated cachexia and other types of disease-associated cachexia. Ghrelin regulates metabolic balance and may improve the cachetic condition through IGF-dependent and IGF-independent pathways. The wide distribution of GHS-R1a suggests multiple roles of ghrelin. Ghrelin and GHS-R1a mediate anti-inflammatory signals in several cell types [63, 64] and in various murine models of acute and chronic inflammation [65]. The vagus nerve is an important link between the nervous system and inflammation. It conveys the immunologic state of the gastrointestinal tract to the hypothalamus. Presence of GHS-R1a in afferent neurons of the nodose ganglion suggests that ghrelin signals are transmitted to the brain through vagal afferent nerves [66]. Ghrelin downregulates IL-6 and TNF*α* in sepsis via the activation of vagus nerve [67]. Ghrelin and its analogue increased food intake and improved lean body mass in cancer rats [68]. Ghrelin treatment in uremia results in improved lean mass accrual in part due to suppressed muscle proteolysis and possibly related to anti-inflammatory effects. Ghrelin-treated nephrectomized rats had a decrease in the expression of circulating inflammatory cytokines, IL-1 receptor and pro-hormone convertase-2, an enzyme involved in the processing of pro-opiomelanocortin to the anorexigenic peptide alpha-MSH [69]. A single subcutaneous injection of ghrelin (3.6 nmol/kg) enhanced short-term (3-day) food intake in a cohort of 9 adult CKD patients [70]. Subsequent report from the same group indicated that daily administration of synthetic ghrelin (12 mcg/kg) stimulated food intake among 12 dialysis patients over a period of 7 days [71]. Importantly, energy expenditure was unchanged and there was no subsequent compensatory reduction in energy intake in these patients. 

Given the complexity of the CKD-associated cachexia, an integrated treatment approach is probably necessary. Although appetite stimulants (e.g., megestrol acetate) showed moderate success in improving patients' appetites [72], two short-term randomized, placebo-controlled trials on ghrelin have renewed hope for the successful treatment of uremic anorexia and wasting [70, 71]. In addition, short-term administration of ghrelin and its analogues is successful in treating several types of disease-associated cachexia [60, 68, 69] and increases lean body mass in healthy adults [42]. Although this new therapeutic approach holds promise, however, several shortcomings and unanswered questions need to be considered. Firstly, the studies were of short duration and the long-term benefits of ghrelin and its analogues in CKD are unknown. We do not know whether the reported improvement in appetite translates into an improved nutritional status and a better outcome. Secondly, long-term ghrelin therapy in CKD may eventually lead to resistance to the effects of ghrelin. Thirdly, given the reported mitogenic potential of ghrelin [73], the possible role of ghrelin in carcinogenesis needs attention. Another concern that might diminish the enthusiasm for long-term ghrelin treatment is the recent report that ghrelin infusion induces lipolysis and insulin resistance [74]. Furthermore, weight gain following ghrelin treatment is associated with gain of fat mass without any change in lean body mass [75]. In vitro experiments indicate that ghrelin stimulates the differentiation of pre-adipocytes and antagonizes adipogenesis, suggesting that ghrelin acts on adipocytes to promote adipogenesis [76]. In summary, small-scale clinical trials have provided encouraging evidence on the short-term orexigenic effects of subcutaneous ghrelin administration in CKD patients. However, the clinical utility of ghrelin in CKD will depend on the long-term outcomes in improving appetite, muscle mass, and function as well as morbidity and mortality. 
